# Supplementary material for: Colored sticky traps for monitoring phytophagous thrips (Thysanoptera) in mango agroecosystems, and their impact on beneficial insects
Source: PLoS One. 2022 Nov 3;17(11):e0276865. doi: 10.1371/journal.pone.0276865 (PMC9632929; doi:10.1371/journal.pone.0276865)
Supplement: S1 Table — Total numbers of thrips captured on colored sticky traps throughout the flowering period of Ataulfo mango. Figures in each sampling represent the thrips captured in six treatments (colors) with eight replicates each. (DOCX) [file pone.0276865.s001.docx]

| **S1 Table. Thrips captured on colored sticky traps** | | | | |
| --- | --- | --- | --- | --- |
| Sampling | *Frankliniella* | *Scirtothrips* | Other thrips | Larvae |
| 1 | 2,348 | 91 | 45 | 56 |
| 2 | 2,712 | 394 | 63 | 56 |
| 3 | 1,555 | 131 | 34 | 4 |
| 4 | 397 | 52 | 39 | 6 |
| 5 | 1,142 | 87 | 53 | 34 |
| 6 | 4,644 | 203 | 34 | 26 |
| 7 | 1,017 | 301 | 85 | 4 |
| 8 | 525 | 203 | 96 | 4 |
| Total | 14,340 | 1,462 | 449 | 190 |
