# Supplementary material for: Colored sticky traps for monitoring phytophagous thrips (Thysanoptera) in mango agroecosystems, and their impact on beneficial insects
Source: PLoS One. 2022 Nov 3;17(11):e0276865. doi: 10.1371/journal.pone.0276865 (PMC9632929; doi:10.1371/journal.pone.0276865)
Supplement: S2 Table — Total numbers of Frankliniella thrips collected from mango inflorescences in seven samplings throughout the flowering period of Ataulfo mango. Each figure in the last column represents the total number of thrips collected from 20 mango inflorescences (10 before traps and 10 after traps). (DOCX) [file pone.0276865.s002.docx]

| **S2 Table. Thrips collected from mango inflorescences** | | | | | | | |
| --- | --- | --- | --- | --- | --- | --- | --- |
| Sampling | Before sticky traps | | | After sticky traps | | | Total |
|  | Larvae | Adults | L + A | Larvae | Adults | L + A |  |
| 1 | 24,541 | 19,295 | 43,836 | 98,762 | 11,210 | 109,972 | 153,808 |
| 2 | 19,140 | 3,795 | 22,935 | 27,433 | 11,447 | 38,880 | 61,815 |
| 3 | 11,842 | 6,960 | 18,802 | 17,595 | 6,553 | 24,148 | 42,950 |
| 4 | 13,621 | 2,945 | 16,566 | 31,991 | 3,516 | 35,507 | 52,073 |
| 5 | 24,573 | 5,841 | 30,414 | 37,275 | 7,340 | 44,615 | 75,029 |
| 6 | 9,921 | 5,659 | 15,580 | 4,470 | 10,622 | 15,092 | 30,672 |
| 7 | 10,334 | 1,430 | 11,764 | 10,560 | 681 | 11,241 | 23,005 |
| Total | 113,972 | 45,925 | 159,897 | 228,086 | 51,369 | 279,455 | 439,352 |
