# Supplementary material for: Colored sticky traps for monitoring phytophagous thrips (Thysanoptera) in mango agroecosystems, and their impact on beneficial insects
Source: PLoS One. 2022 Nov 3;17(11):e0276865. doi: 10.1371/journal.pone.0276865 (PMC9632929; doi:10.1371/journal.pone.0276865)
Supplement: S3 Table — Absolute abundance of natural enemies (parasitoids and predators) on colored sticky traps in Ataulfo mango agroecosystems. (DOCX) [file pone.0276865.s003.docx]

| **S3 Table. Natural enemies** | | | | | | |
| --- | --- | --- | --- | --- | --- | --- |
| Order: Family | Blue | Green | Orange | Purple | White | Yellow |
| Araneae | 120 | 131 | 141 | 133 | 141 | 139 |
| Coleoptera | 37 | 41 | 49 | 40 | 52 | 67 |
| Anthicidae | 0 | 1 | 0 | 0 | 0 | 0 |
| Carabidae | 2 | 5 | 7 | 5 | 11 | 4 |
| Coccinellidae | 10 | 15 | 19 | 9 | 6 | 40 |
| Derodontidae | 0 | 0 | 0 | 0 | 1 | 0 |
| Histeridae | 1 | 0 | 2 | 0 | 1 | 0 |
| Hydrophilidae | 0 | 1 | 0 | 0 | 0 | 0 |
| Noteridae | 0 | 0 | 0 | 0 | 1 | 0 |
| Rhizophagidae | 0 | 0 | 0 | 0 | 1 | 0 |
| Staphylinidae | 24 | 19 | 21 | 26 | 31 | 23 |
| Diptera | 10 | 14 | 28 | 19 | 10 | 65 |
| Dolichopodidae | 9 | 8 | 24 | 18 | 10 | 62 |
| Pipunculidae | 0 | 2 | 0 | 0 | 0 | 0 |
| Scenopinidae | 0 | 1 | 0 | 0 | 0 | 0 |
| Tachinidae | 1 | 3 | 4 | 1 | 0 | 3 |
| Hemiptera | 7 | 12 | 5 | 14 | 15 | 9 |
| Anthocoridae | 6 | 8 | 4 | 11 | 9 | 5 |
| Enicocephalidae | 1 | 3 | 1 | 3 | 5 | 2 |
| Reduviidae | 0 | 1 | 0 | 0 | 1 | 2 |
| Hymenoptera | 360 | 568 | 538 | 289 | 301 | 711 |
| Aphelinidae | 38 | 62 | 42 | 24 | 24 | 53 |
| Bethylidae | 15 | 24 | 20 | 6 | 9 | 32 |
| Braconidae | 1 | 6 | 5 | 1 | 4 | 6 |
| Ceraphronidae | 13 | 22 | 26 | 27 | 15 | 31 |
| Chalcididae | 0 | 5 | 3 | 1 | 0 | 5 |
| Diapriidae | 3 | 2 | 1 | 1 | 2 | 4 |
| Elasmidae | 0 | 2 | 0 | 1 | 0 | 0 |
| Encyrtidae | 60 | 79 | 72 | 45 | 50 | 169 |
| Eulophidae | 25 | 22 | 25 | 9 | 17 | 15 |
| Eupelmidae | 0 | 1 | 0 | 4 | 0 | 1 |
| Eurytomidae | 1 | 1 | 1 | 0 | 0 | 0 |
| Figitidae | 0 | 5 | 1 | 0 | 2 | 0 |
| Ichneumonidae | 0 | 0 | 0 | 1 | 0 | 2 |
| Mymaridae | 77 | 119 | 116 | 94 | 81 | 108 |
| Platygastridae | 2 | 9 | 14 | 5 | 5 | 20 |
| Pteromalidae | 7 | 24 | 12 | 1 | 3 | 20 |
| Scelionidae | 86 | 132 | 156 | 52 | 57 | 197 |
| Signiphoridae | 8 | 15 | 18 | 7 | 12 | 15 |
| Trichogrammatidae | 24 | 38 | 26 | 10 | 20 | 33 |
| Neuroptera | 0 | 1 | 2 | 1 | 2 | 0 |
| Chrysopidae | 0 | 0 | 1 | 0 | 0 | 0 |
| Coniopterygidae | 0 | 1 | 1 | 1 | 1 | 0 |
| Mantispidae | 0 | 0 | 0 | 0 | 1 | 0 |
| Thysanoptera | 34 | 36 | 41 | 36 | 22 | 20 |
| Aeolothripidae  *Stomatothrips flavus* | 1 | 4 | 2 | 1 | 2 | 2 |
| Thripidae  *Scolothrips pallidus* | 1 | 3 | 1 | 2 | 0 | 3 |
| Phlaeothripidae  *Karnyothrips texensis* | 32 | 29 | 38 | 33 | 20 | 15 |
| Total | 568 | 803 | 804 | 532 | 543 | 1,011 |
