# Supplementary material for: Colored sticky traps for monitoring phytophagous thrips (Thysanoptera) in mango agroecosystems, and their impact on beneficial insects
Source: PLoS One. 2022 Nov 3;17(11):e0276865. doi: 10.1371/journal.pone.0276865 (PMC9632929; doi:10.1371/journal.pone.0276865)
Supplement: S4 Table — Absolute abundance of mango insect pollinators captured with colored sticky traps in Ataulfo mango agroecosystems. (DOCX) [file pone.0276865.s004.docx]

| **S4 Table. Mango insect pollinators** | | | | | | |
| --- | --- | --- | --- | --- | --- | --- |
| Order: Family | Blue | Green | Orange | Purple | White | Yellow |
| Coleoptera | 0 | 1 | 1 | 0 | 1 | 0 |
| Cantharidae | 0 | 1 | 1 | 0 | 1 | 0 |
| Diptera | 114 | 172 | 159 | 87 | 62 | 107 |
| Chironomidae | 13 | 15 | 17 | 16 | 17 | 20 |
| Chloropidae | 21 | 23 | 24 | 11 | 9 | 21 |
| Milichiidae | 27 | 40 | 49 | 15 | 10 | 19 |
| Sciaridae | 53 | 93 | 65 | 44 | 26 | 44 |
| Tabanidae | 0 | 0 | 1 | 0 | 0 | 0 |
| Tipulidae | 0 | 1 | 3 | 1 | 0 | 3 |
| Hymenoptera | 27 | 34 | 29 | 29 | 35 | 17 |
| Agaonidae | 8 | 3 | 4 | 7 | 3 | 4 |
| Apidae | 1 | 0 | 0 | 1 | 1 | 1 |
| Formicidae | 16 | 30 | 25 | 21 | 31 | 11 |
| Halictidae | 1 | 1 | 0 | 0 | 0 | 0 |
| Vespidae | 1 | 0 | 0 | 0 | 0 | 1 |
| Total | 141 | 207 | 189 | 116 | 98 | 124 |
