# Supplementary material for: Colored sticky traps for monitoring phytophagous thrips (Thysanoptera) in mango agroecosystems, and their impact on beneficial insects
Source: PLoS One. 2022 Nov 3;17(11):e0276865. doi: 10.1371/journal.pone.0276865 (PMC9632929; doi:10.1371/journal.pone.0276865)
Supplement: S5 Table — Means of Frankliniella thrips in seven samplings throughout the flowering period of Ataulfo mango in Chiapas, Mexico. Figures of each sampling represent the average specimens collected in 20 mango inflorescences. (DOCX) [file pone.0276865.s005.docx]

| **S5 Table. Thrips catches** | | | |
| --- | --- | --- | --- |
| Sampling | Larvae | Adults | Larvae + Adults |
| 1 | 6,165.15 | 1,525.25 | 7,690.40 |
| 2 | 2,328.65 | 762.10 | 3,090.75 |
| 3 | 1,471.85 | 675.65 | 2,147.50 |
| 4 | 2,280.60 | 323.05 | 2,603.65 |
| 5 | 3,092.40 | 659.05 | 3,751.45 |
| 6 | 719.55 | 814.05 | 1,533.60 |
| 7 | 1,044.70 | 105.55 | 1,150.25 |
| Total Avg. | 2,443.27 | 694.96 | 3,138.23 |
