# Supplementary material for: Colored sticky traps for monitoring phytophagous thrips (Thysanoptera) in mango agroecosystems, and their impact on beneficial insects
Source: PLoS One. 2022 Nov 3;17(11):e0276865. doi: 10.1371/journal.pone.0276865 (PMC9632929; doi:10.1371/journal.pone.0276865)
Supplement: Attachment — Submitted filename: REVIEW OUTLINE.docx [file pone.0276865.s007.docx]

**REVIEW OUTLINE**

Summary of the research:

the submitted article PLOSE-D-22-14742 titled “ Colored sticky traps for monitoring phytophagous thrips (Thysanoptera) in mango agrosystems and their impact on beneficial insects”, is articulated in the following paragraphs : 1)Introduction,2) Material and Methods, 3) Results, 4) Discussion, 5) Conclusions and 6) Reference List. Paragrapf 1 provides sufficient background and includes all proper references which are listed in paragraph 7. In M & M (2) all the applied methods and techniques in each step of the research design are clearly described, even through some parts need to be clarified or improved in technical details (see my notes/ suggestions in the attached word file of the text). Reading the manuscript, I noted that the main questions proposed by the research have been: are the coloured sticky traps useful tools to collect the thrips species and to evaluate biodiversity of beneficial insects (pollinators and natural enemies) of the studied agroecosystem (mango crop)? Can the colour trap efficiency be determined in attracting pest thrips on the target crop and can be evaluated the relationships of the thrips abudance on traps and that one observed in the inflorescences? The results (3) obtained showed that the white traps are the most attractive for *Frankliniella* species – during mango flowering, instead yellow traps are more attractive for Scirtothrips species- after setting of fruits. Moreover, the ratio between the thrips density on traps and that one into inflorescences is positive but not significant.

The main claims refferred to the results are the great amount of field data obtaned only for a crop (mango) and the establishing of the most important period of the plant biology useful to apply the traps to permit the most abudant captures of pest thrips. The conclusions assert these results and also that the use of colored sticky traps would be a good option for monitoring mango thrips in earlier stages of infestation to implement management methods and to avoid the building-up of thrips populations. Also the choice of some colored traps useful to collect natural enemies or pollinators (both identified only at Family level) constitutes a valid tool provided in order to verify biodiversity of beneficial insect associated to the agrosystem. Finally, the major issues I would to address in order to proceed of the manuscript are:

1) The molecular identification of the caught pest thrips species belonging to *Frankliniella* (*invasor*, *gardeniae*, *cephalica*) and *Scirtothrips* ( *citri*, *manihoti*) genera. The lack penalizes , on scientific base, this study because the mentioned species are difficult to identify through the morphological character states provided by the cited taxonomic literature. So that, a combination between morpho-taxonomy and DNA barcode/ sequencing could be suitable;
2) Improvement of statistical analysis of the field data for pest thrips captured on colored sticky traps and those observed in mango inflorescences. The analysis should refer only the most abundant pest species collected, i.e. *Frankliniella invasor*, in order to establish a significant correlation between the two screens. This relationships request to improve sampling methods to reduce the sampling variability and increase the degree of correlation.
