# Supplementary material for: Colored sticky traps for monitoring phytophagous thrips (Thysanoptera) in mango agroecosystems, and their impact on beneficial insects
Source: PLoS One. 2022 Nov 3;17(11):e0276865. doi: 10.1371/journal.pone.0276865 (PMC9632929; doi:10.1371/journal.pone.0276865)
Supplement: Attachment — Submitted filename: Response to Reviewers [12 October 2022].docx [file pone.0276865.s009.docx]

**“Colored sticky traps for monitoring phytophagous thrips (Thysanoptera) in mango agroecosystems, and their impact on beneficial insects”**

**[**PONE-D-22-14742**]**

Response to Reviewers

We appreciate the positive and constructive comments made by the reviewers. Below, please find our responses**.**

***Reviewer #1:*** *I accept nearly all the responses of the authors. I have a few minor comments that should be easy to fix.*

*I pointed out that the reflectance in the UV from 350-400 nm is very important for thrips responses. The authors say that the spectrophotometer readings did in fact go down to 350 nm. However, Fig. 2 appears to show readings down to only about 375 nm. If the data exist for wavelengths down to 350 nm, these should be included by extending the wavelength axis to show down to 350 nm. However, if the data for 350 nm are not shown because they are unreliable below 375 nm, it should be stated that measurements went down to 375 nm. At the moment it seems odd to state that the measurements were made at the critical wavelengths down to 350 nm but then to omit them from Fig. 2.*

**Response:** Even though our spectrometer provided readings down to 350nm the readings between 350 and 375nm were noisy and not reliable, therefore the reliable range of 375nm to 1000nm was considered for this work. This issue has been clarified in the material and methods section.

***Reviewer #1:*** *I am still not clear about the units for the axes labelled as “number of individuals” or “thrips per trap” (Figs. 3, 4, 6). Are these all* *catches per 72 hours or have* *catches from several sampling dates been combined? What is the timespan of these catches? The rate of catch is of interest. This could be clarified easily by adding this in the figure legend if it does not fit on the axis.*

**Response:** We have added the units for each axis in the figure legends to clarify this issue.

***Reviewer #1:*** *Line 222. The authors responded that the negative binomial distribution was assumed and assessed. It would reassure the statistically interested reader if it could be stated that the distribution was not just assessed but also confirmed or found to fit well. The fit could have been assessed and found to fit badly.*

**Response:** We have revised and modified this phrase in the text as suggested.

***Reviewer #1:*** *The paper repeatedly mentions monitoring and investigates whether trap catches correlate with relative abundance, which would allow abundance monitoring. The results showed no significant correlation between trap catch and abundance in inflorescences, but concludes that traps would be useful for monitoring. The authors state that traps would be “a good option for monitoring mango thrips in earlier stages of infestation”. What I think is confusing is that the term “monitoring” is used through most of the paper in the context of measuring abundance but then uses it, without being explicit, to mean monitoring for detection rather than monitoring for abundance. I think it would be clearer to the reader if detection was made explicit. For example, the above sentence could say something like* *“a good option for monitoring mango thrips to detect them at earlier stages of infestation”*

**Response:** The context of the word ‘monitoring’ has been certainly stated in lines 86-90. However, we have modified the phrase to satisfy the reviewer.

***Reviewer #1:*** *I remain dubious about the statistical test to show that the three colors with peaks in the shorter wavelengths caught more thrips than the three colors that did not have peaks in the shorter wavelengths. This appears to be an a posteriori hypothesis. In other words, it appears that the authors looked for wavelengths that occurred in the colours that caught more and then tested this statistically on the data that had just suggested the hypothesis. Such a hypothesis is interesting but the statistical test that has been used is for an a priori hypothesis. However, I think that there is enough information there for the reader to draw their own conclusions.*

**Response:** These are post-hoc linear contrasts which are routinely used with statistical models and we are doing nothing different here. We followed standard practice: 1) is the model better than null model; 2) if so, are any factors significant; 3) if so, run post hoc comparisons to understand those relationships (e.g. the whole field of LSM/EMMEANS). We can understand the reviewer's point that one can argue about how the p-value should be adjusted since we also ran separate pairwise comparisons. However, the conclusions are robust (very low p-value) and we provide the 95%CI to support the strength of the relationships. The reviewer suggests not including support for these statements, but we argue stating that the shorter wavelength differed without any stat support (e.g. estimate with uncertainty) is rather handwavy and not convincing. Even the reviewer says that the readers will make the conclusion themselves, so why not provide the readers with the estimate and a measure of the uncertainty in that estimate?

***Reviewer #2:*** No corrections were suggested by this reviewer

Thanks!
